# Attention deficit hyperactivity disorder is associated with (a)symmetric tonic neck primitive reflexes: a systematic review and meta-analysis

**DOI:** 10.3389/fpsyt.2023.1175974

**Published:** 2023-07-07

**Authors:** Meng Wang, Jing Yu, Hyun-Duck Kim, Angelita Bautista Cruz

**Affiliations:** ^1^Department of Physical Education, Keimyung University, Daegu, Republic of Korea; ^2^College of Sports Science, Shenyang Normal University, Shenyang, China; ^3^Department of Sport Marketing, Keimyung University, Daegu, Republic of Korea

**Keywords:** primitive reflexes, attention deficit and hyperactivity disorder, meta-analysis, asymmetric tonic neck reflex, symmetric tonic neck reflex

## Abstract

**Introduction:**

Investigation on the association between attention deficit hyperactivity disorder (ADHD) and primary reflexes is in the initial stage, with considerable differences in the findings. This study evaluated the association between ADHD and primitive reflexes using systematic review and meta-analysis.

**Methods:**

Data were obtained from PubMed, Cochrane Library, Web of Science, EBSCO (Medical Literature Analysis and Retrieval System Online, American Psychological Association Psyclnfo, and Education Resources Information Center), Embase, Scopus, and ProQuest. Articles were searched from the date of inception of the respective databases to January 01, 2023, and StataCorp Stata (version 15) was used for the analysis.

**Results:**

Four articles with 229 samples were included in the meta-analysis. Results showed a significant positive and moderate correlation between ADHD and primitive reflexes, particularly asymmetric tonic neck reflex: summary *r* value = 0.48, 95% CI = 0.27–0.64; symmetric tonic neck reflex: summary *r* value = 0.39, 95% CI = 0.25–0.52. Overall, findings from the sub-group analysis indicate that the behavioral problem measuring tool (Conners’ scale), sex, and primitive reflex test could significantly moderate the relationships between ADHD and ATNR and STNR primitive reflexes.

**Conclusion:**

ADHD symptoms in children are closely related to the non-integration of (a)symmetric tonic neck primitive reflexes. Longitudinal or experimental studies should be conducted to reveal the causal relationship between ADHD and primitive reflexes in the future.

## 1. Introduction

Attention deficit hyperactivity disorder (ADHD) is the most common neurodevelopmental disorder in children, with a global prevalence of approximately 5.2–7.2% (1). Approximately 65% of ADHD symptoms persist into adulthood (2). ADHD can result in emotional disorders, learning disorders, social relations, and social adaptation disorders. It is also significantly related to comorbid mental illnesses, including personality disorders, self-harm, and drug abuse (3–5). Furthermore, it exerts several negative effects on several aspects of life and is an important public health concern (6). Interestingly, these ADHD symptoms are found to have similarities with retention of primitive reflexes or when survival-oriented and automatic movements during an infant’s developmental stage are not normally suppressed/integrated (7).

Primitive reflexes occur during the fetal or infant period. Infants demonstrate more than 20 types of primitive reflexes, including swallowing, breathing, audiovisual, head movement, hand grasping, trunk control, and lower limb movement (8, 9). These reflexes are involuntary responses to external stimuli to protect the body. During development, most primitive reflexes gradually integrate and modify into more complex patterns, achieving self-control movements and skills (9). Only a small part of them persists, such as the blink reflex and swallowing reflex. However, if the primitive reflex is not integrated beyond the normal 3-year development period, it will exert a negative impact on the developmental process in children and reduce the brain’s ability to effectively process sensory information (10). Children who have not integrated these reflexes are more likely to experience social inclusion issues. They demonstrate a lower ability to interact with other children, greater anxiety, stronger resistant behavior, and a reserved personality than those with integrated primitive reflexes, thereby making it difficult to establish good interpersonal relationships (11–14). Further, children with retained primitive reflexes demonstrate difficulties in controlling their body positioning and movements, resulting in problems with motor coordination, such as wrist lifting, finger extension, and foot positioning (15–17). Furthermore, they are prone to hyperactivity or impulsivity, particularly in tasks that require cognitive effort or long periods of concentration. This is because they easily lose focus, leading to learning difficulties (7, 18). While there are several known primitive reflexes, this research is focused on asymmetric tonic neck reflex (ATNR) (16–18) and symmetric tonic neck reflex (STNR) (16–18). ATNR is a primitive reflex when an infant extends the arm and leg on the facial side and flexes the opposite leg and arm resulting from turning the baby’s head to one side. This reflex appears in the uterus at approximately 18 weeks and integrates 3 to 9 months after birth (9, 19). Meanwhile, STNR is when an infant flexes the head, it prompts flexion and extension of the arms and legs, respectively. Conversely, when an infant extends the head, it elicits extension of the arms and flexion of the legs. This reflex appears approximately 6 to 9 months after birth and integrates 9 to 11 months after birth (9, 19). The lack of ATNR integration may lead to difficulties in processing fine and gross motor tasks, whereas the lack of STNR integration leads to poor organizational ability, difficulty in hand-eye coordination, and abnormal or “ape-like” gait (19). These atypical behaviors often appear in children diagnosed with ADHD (19).

Studies have demonstrated similarities in behavioral and cognitive problems between non-integrated primitive reflexes and ADHD because their common and partial symptoms are either similar or overlapping (7, 9, 20). Sensory and motor dysfunctions can result from primitive reflexes that are not integrated within a reasonable period in children; these dysfunctions are also evident in children with ADHD (21). In addition, patients with several neurodevelopmental disorders, including autism (22), bipolar affective disorder (23), dyskinesia cerebral palsy (24, 25), and dyslexia (22), report problems in the integration of primitive reflexes. These issues will exert a profound negative impact on the lives of children, and, therefore, should be promptly identified such that appropriate interventions or treatments are provided to reduce or eliminate their undesirable consequences.

Currently, research on the correlation between ADHD and primary reflexes is in the preliminary stage, and there are large differences in the results. Therefore, we adopted a more accurate and comprehensive meta-analysis approach to analyze and explore the regulatory variables based on the relationship between ADHD and primitive reflexes. This approach can avoid the deviation of the results caused by the influence of sample size, sex, and other factors in a single study, is conducive to draw more accurate and general conclusions from a macroperspective, and proposes directions for future research.

## 2. Methods

A meta-analysis was conducted and reported according to the preferred reporting items of the system evaluation and meta-analysis guidelines. The scheme has been registered at the (26) International Platform of Registered Systematic Review and Meta-analysis Protocols (IMPLASY) with the registration number IMPLASY202310022.^1^

### 2.1. Literature retrieval strategy

We searched the following bibliographic databases from their inception to January 01, 2023, without restrictions on the language or publication year: PubMed, Cochrane Library, Web of Science, EBSCO [Medical Literature Analysis and Retrieval System Online (MEDLINE), American Psychological Association (APA) Psyclnfo, and Education Resources Information Center (ERIC)], Embase, Scopus, and ProQuest. Only studies published in English were included in this meta-analysis. In addition, the reference lists of the included studies and systematic reviews of primitive reflex movements in children with ADHD from the last 10 years were further scrutinized to identify other relevant studies. The search keywords included “attention deficit disorders with hyperactivity,” “primitive reflexes,” and “children.” Retrieval combined keywords and free words, which were determined by repeated prechecks and supplemented by the manual retrieval of gray literature. Language and publication types were not limited during retrieval.

### 2.2. Inclusion and exclusion criteria

#### 2.2.1. Inclusion criteria

The following studies were included in this systematic review and meta-analysis: (1) studies that cleared the diagnosis of ADHD symptoms, i.e., the Diagnostic and Statistical Manual of Mental Disorders DSM-IV (27) DSM-5 (28), (2) studies that cleared primitive reflex evaluation criteria, i.e., INPP Reflex Assessments Test (29), Bender-Purdue Reflex Test (30), Schilder test (31), (3) studies that reported on specific data (such as the correlation coefficient and sample size) of ADHD and primary reflexes without evident errors, and (4) studies that included participants aged <18 years.

#### 2.2.2. Exclusion criteria

The following studies were excluded from this systematic review and meta-analysis: (1) studies that included participants diagnosed with dementia and mental health disorders; (2) studies with repeated publication or poor-quality evaluation; and (3) reviews, surveys, and conference or case reports.

### 2.3. Data extraction and coding

Two of the authors independently extracted information from the literature. In case of differences in the subsequent cross-check, the remaining authors were consulted for adjudication. The extraction and coding contents included the following aspects: (1) basic information of the literature, namely, the author, publication year, nationality, r value, and main conclusion; (2) basic information of the study participants, namely, the age, sample size, and sampling method.

### 2.4. Quality evaluation

The bias risk assessment tool (32) developed by Hoy et al. was used for a comprehensive evaluation of the bias risk. The tool comprises 10 items as follows: four items are related to the external effectiveness of the sample representative of the study population, and six items are related to the internal effectiveness of the study. The total mass fraction of each study was obtained by summing the binary reaction fractions (yes = 1 or no = 0) ranging from 0 to 10. Scores >7 and between 6 and 7 indicated low and moderate risks of bias, respectively. A score < 6 indicated a high risk of bias.

### 2.5. Statistical method

The included studies had different correlation analysis models. To ensure the accuracy of the results, we converted the Pearson correlation coefficient into the Spearman correlation coefficient, which was implemented in the WPS software. The conversion formula was as follows:


$$r_{s}=\frac{6}{\pi }\sin^{-1}\frac{r_{p}}{2};$$

where *r_s_* is the Spearman correlation coefficient and *r_p_* is the Pearson correlation coefficient. The statistics that ADHD and primitive reflex correlation coefficients were not reported, but *t*, *F*, and $\chi^{2}\;$ were reported were converted to r (33). The conversion formula was as follows:


$$r=\sqrt{t^{2}/t^{2}+df},\;\;\;\;\;\;df=n_{1}+n_{2}-2;$$

$$r=\sqrt{F\left(1,-\right)/F\left(1,-\right)+df\left(\text{error}\right)\;};$$

$$r=\sqrt{\chi^{2}/\chi^{2}+N};$$

If the summary r value is calculated by combining the correlation coefficient r values of each study, the result will deviate significantly. Therefore, it was necessary to convert the r value of each literature to the Fisher’s Z value, calculate the summary Fisher’s Z value by adding each Fisher’s Z value from relevant studies, and subsequently convert the summary Fisher’s Z value to summary r value. Finally, the summary r value was the correlation coefficient after combining the relevant studies. We used the software Stata 15 for the analysis, data conversion, effect amount consolidation, sensitivity analysis, and subgroup analysis. If *p* > 0.1 and *I*^2^ < 50%, similar studies can be considered homogeneous, and a fixed-effects model can be selected; if *p* < 0.1 and *I*^2^ ≥ 50%, a random-effects model can be selected. When the heterogeneity was large, we combined subgroup and sensitivity analyzes to determine the source of heterogeneity and evaluate the stability of the results. Egger’s test was performed to evaluate publication bias. Finally, the correlation was based on the converted summary r value. After considering the absolute value of r, values ranging from 0–0.09, 0.1–0.3, 0.3–0.5, and 0.5–1.0 were considered extremely weak, weak, moderate, and strong correlations, respectively (34).

## 3. Results

### 3.1. Literature retrieval

**Figure 1:**
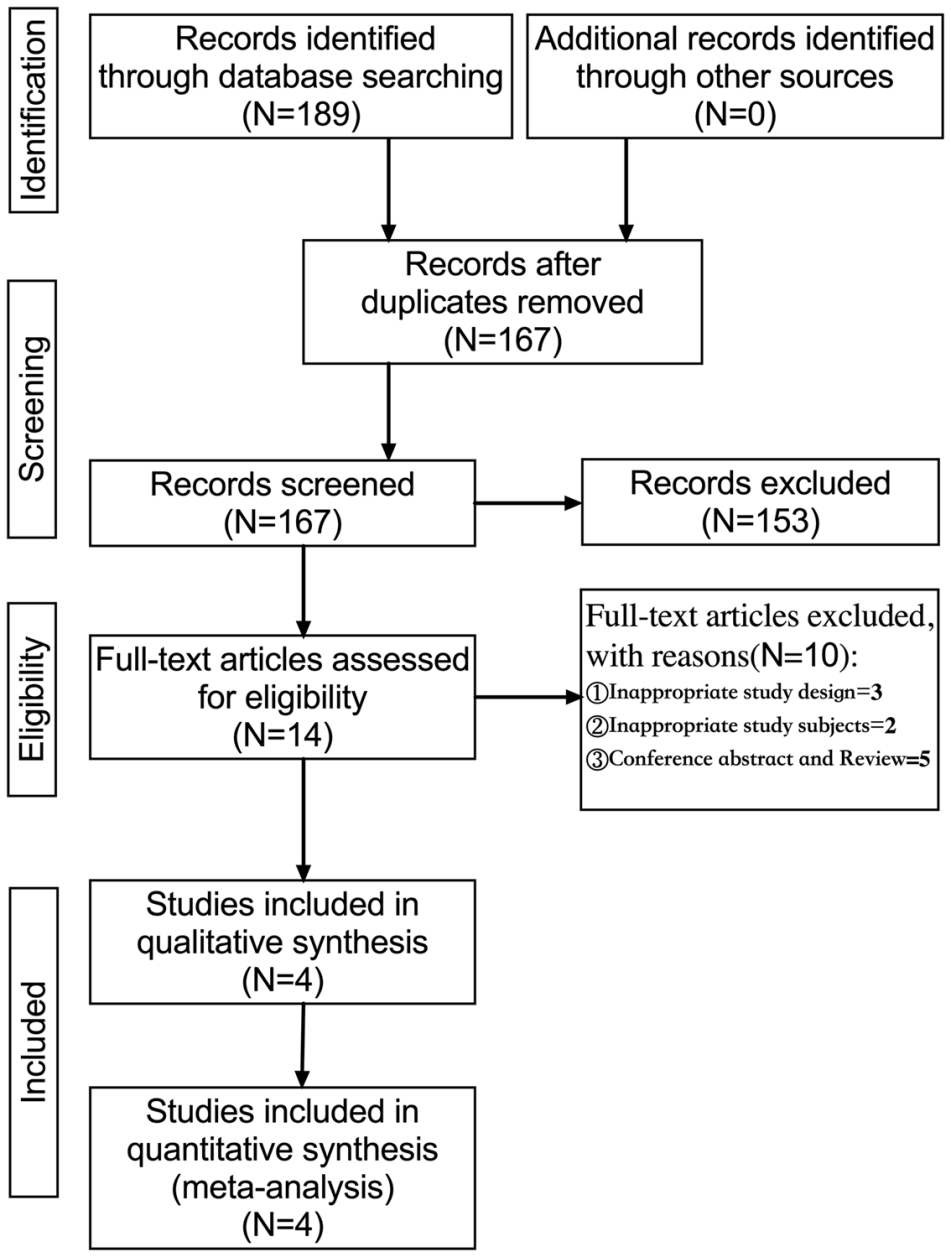
Flowchart of the study selection process.

A total of 189 articles were identified by searching PubMed (*n* = 5), Cochrane Library (*n* = 1), Web of Science (*n* = 4), EBSCO (MEDLINE, APA Psyclnfo, and ERIC; *n* = 3), Embase (*n* = 57), Scopus (*n* = 118), and ProQuest (*n* = 1). These articles were imported into EndNote X9, and 167 articles were obtained after deduplication. Fourteen articles were initially screened by reading their titles and abstracts, and four were selected for quantitative synthesis (meta-analysis; Figure 1).

### 3.2. Characteristics and quality evaluation of the included literature

**Figure 2:**
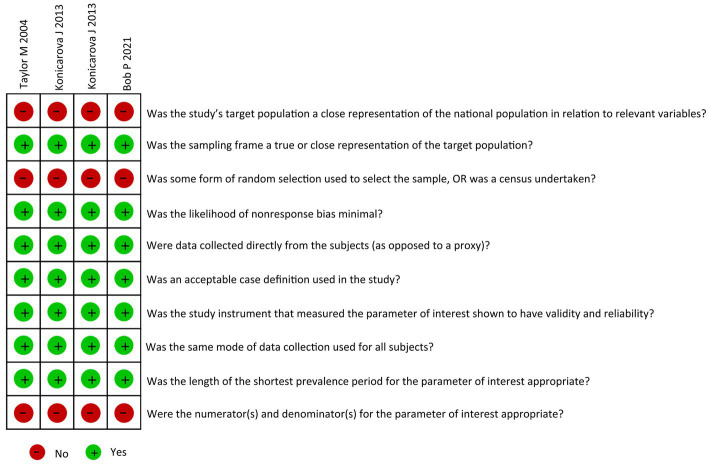
Risk of bias.

A preliminary search was conducted for 189 articles, and four articles were selected comprising five studies (Table 1). The study designs were as follows: Spearman correlation analysis model (*n* = 3) and Pearson correlation analysis model (*n* = 1). A total of 229 patients with ADHD were assessed, and primitive reflex evaluations were performed as follows: ATNR (*n* = 5) and STNR (*n* = 4). These four articles had a low risk of bias (Figure 2).

**Table 1:** Basic characteristics of the included articles.

Study	Year	Country	Diagnostic criteria	*n*	Measurement of primitive reflex	Age (mean)	Test scale	*r*		Main conclusion
								Primitive reflexes		
								ATNR	STNR	
Taylor et al. (7)	2004	Australia	DSM-IV	54	INPP Reflex Assessments Test	9.32 (8–11)	CPRS-R	0.2680	0.3820	ADHD was associated with primitive reflexes
Konicarova et al. (18)	2013	Czech Republic	DSM-IV	35	Schilder Test and Bender– Purdue Reflex Test	9.51 (8–11)	CPQ	0.6607	0.4271	ADHD symptoms were associated with persistent ATNR and STNR
Konicarova and Bob (16)	2013	Czech Republic	DSM-IV	60	Schilder test	9.32 (8–11)	CPQ	0.6081	Null	ADHD symptoms were closely related to persistent ATNR
Bob et al. ① (17)	2021	Czech Republic	DSM-IV	40	Schilder Test and Bender–Purdue Reflex Test	9.21 (8–11)	CPQ	0.5680	0.240	ADHD symptoms were closely associated with persisting ATNR
Bob et al. ② (17)	2021	Czech Republic	DSM-IV	40	Schilder Test and Bender–Purdue Reflex Test	9.30 (8–11)	CPQ	0.1778	0.5176	ADHD symptoms were closely related to persistent STNR

CPQ, Conners’ Parent Questionnaire; CPRS-R, Conners’ parent rating scale-revised; ATNR, asymmetric tonic neck reflex; STNR: Symmetric tonic neck reflex; INPP, Institute for Neuro-Physiological Psychology; and DSM-IV, Diagnostic and Statistical Manual of Mental Disorders, fourth edition.

### 3.3. Results of the meta-analysis

#### 3.3.1. Association between ADHD and ATNR

**Figure 3:**
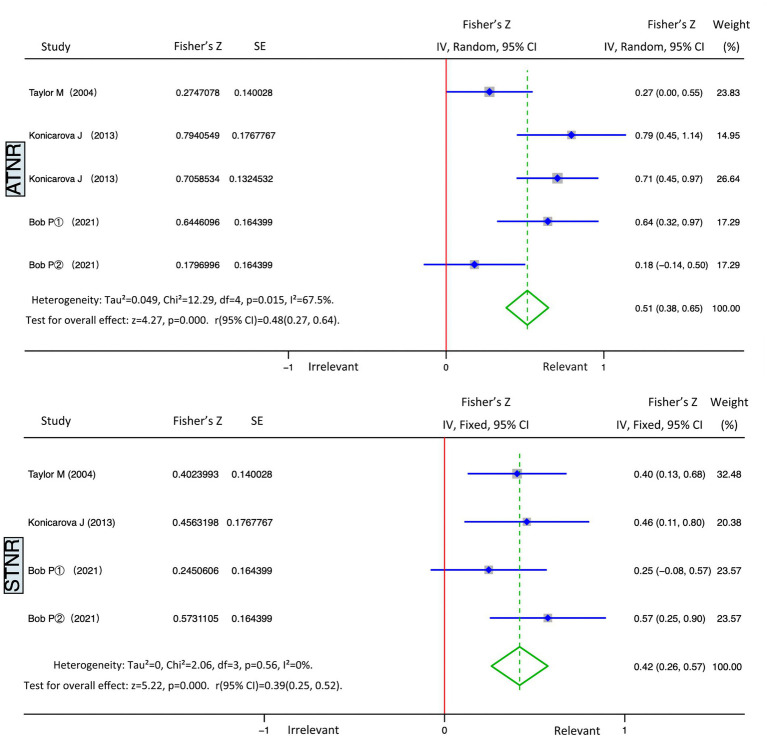
Forest plot of meta-analysis (7, 16–18).

The five studies on ADHD and ATNR had a heterogeneity (*I*^2^) of 67.5% (>50%), and the Q test indicated *p* = 0.015 (<0.05). The studies demonstrated moderate heterogeneity; therefore, random effects were selected for the meta-analysis. We obtained statistically significant results for heterogeneity (Summary Fisher’s Z = 0. 51, 95% CI: 0.38–0.65, *p* = 0.015) and statistical test (Z = 4.27, *p* < 0.01). According to the formula, the summary Fisher’s Z value (0.51) could be converted to a summary r value of 0.48 (95% CI: 0.27–0.64, *p* < 0.01). Results showed that ATNR and ADHD had significant positive and moderate correlation, indicating that the higher the degree of ATNR non-integration, the higher the level of ADHD (Figure 3, top panel).

#### 3.3.2. Association between ADHD and STNR

The four studies on ADHD and STNR had a heterogeneity (*I*^2^) of 0% (<50%), and the Q test indicated *p* = 0.56 (>0.05). These studies demonstrated no heterogeneity; thus, fixed effects were selected for the meta-analysis. We obtained statistically significant results for heterogeneity (Summary Fisher’s *Z* = 0. 42, 95% CI: 0.26–057, *p* = 0.56) and statistical test (*Z* = 5.22, *p* < 0.01). According to the formula, the summary Fisher’s *Z* value (0.42) could be converted to a summary r value of 0.39 (95% CI: 0.25–0.52, *p* < 0.01). Results revealed that STNR and ADHD had significant positive and moderate correlation, indicating that the higher the degree of STNR non-integration, the higher the level of ADHD (Figure 3, bottom part).

#### 3.3.3. Subgroup analysis (Conners’ scale)

To explore whether the heterogeneity among studies was attributed to a single study, we performed a sensitivity analysis using Stata 15 software. We observed no significant change in the effective response rate after eliminating one study sequentially, indicating stable results. To further explore the potential sources of heterogeneity, we conducted a subgroup analysis. All studies included Conners’-type scales, which are widely used to screen behavioral problems in children (particularly ADHD). The Conners’ Parent Questionnaire and Conners’ Parent Rating Scales-Revised are principally used to examine conduct problems, anxiety impulsivity-hyperactivity, learning problems, and perfectionism among children and adolescents aged between 3 and 17 years. These scales have good reliability and validity.

Table 2 summarizes the results of the subgroup analyzes. Results from the five combined studies in the ATNR and conduct problems group demonstrated a summary r value of 0.25 (*p* < 0.01), whereas those from the four combined studies in the STNR and conduct problems group demonstrated a summary r value of 0.28 (*p* < 0.01). Results revealed a significant positive weak correlation of ATNR and STNR with conduct problems. This finding indicates that the higher the degree of ATNR and STNR non-integration, the more severe the conduct problems.

**Table 2:** Subgroup analysis of primitive reflexes and ADHD.

Variable		PR	*n*	Heterogeneity test result			Accumulate correlation coefficient r (95% CI)	Effects model	Effect size test		
				Q	*p*-value	*I*^2^/%			Fisher’s Z (95% CI)	*z*	*p*-value
Conduct problems		ATNR	5	1.77	0.78	0	0.25 (0.12, 0.37)	Fixed	0.26 (0.13, 0.39)	3.78	<0.01
		STNR	4	1.89	0.59	0	0.28 (0.13, 0.41)	Fixed	0.28 (0.13, 0.44)	3.56	<0.01
Anxiety		ATNR	4	2.26	0.52	0	0.33 (0.19, 0.46)	Fixed	0.34 (0.19, 0.50)	4.40	<0.01
		STNR	3	0.58	0.75	0	0.0007 (−0.19, 0.19)	Fixed	0.001 (−0.19, 0.19)	0.008	0.99
Impulsivity-hyperactivity		ATNR	5	4.25	0.37	5.9	0.41 (0.29, 0.52)	Fixed	0.43 (0.30, 0.57)	6.35	<0.01
		STNR	4	0.57	0.90	0	0.36 (0.22, 0.49)	Fixed	0.38 (0.22, 0.53)	4.72	<0.01
Learning problems		ATNR	5	4.26	0.37	6.1	0.25 (0.12, 0.38)	Fixed	0.26 (0.13, 0.40)	3.68	<0.01
		STNR	3	0.28	0.87	0	0.43 (0.26, 0.57)	Fixed	0.46 (0.27, 0.65)	4.70	<0.01
Perfectionism		ATNR	4	0.69	0.88	0	0.39 (0.25, 0.51)	Fixed	0.41 (0.25, 0.56)	5.21	<0.01
		STNR	3	1.32	0.52	0	0.12 (−0.07, 0.30)	Fixed	0.12 (−0.07, 0.31)	1.27	0.20
Muscular tension		ATNR	4	0.45	0.93	0	0.29 (0.15, 0.42)	Fixed	0.30 (0.15, 0.45)	3.83	<0.01
		STNR	3	4.19	0.12	52.3	0.15 (−0.04, 0.34)	Random	0.15 (−0.04, 0.34)	1.08	0.28
Psychosomatic difficulties		ATNR	3	0.01	0.99	0	0.07 (−0.12, 0.25)	Fixed	0.07 (−0.12, 0.26)	0.68	0.49
		STNR	3	0.22	0.90	0	0.04 (−0.15, 0.23)	Fixed	0.04 (−0.15, 0.23)	0.44	0.66
Antisocial behavior		ATNR	3	0.85	0.65	0	0.23 (0.05, 0.40)	Fixed	0.24 (0.05, 0.43)	2.43	0.01
		STNR	3	3.01	0.22	33.5	0.016 (−0.17, 0.20)	Fixed	0.02 (−0.17, 0.21)	0.13	0.89
Sex	Boys	ATNR	2	0.19	0.66	0	0.23 (0.03, 0.42)	Fixed	0.24 (0.03, 0.44)	2.20	0.03
		STNR	2	0.62	0.43	0	0.44 (0.26, 0.59)	Fixed	0.47 (0.27, 0.68)	4.45	<0.01
	Girls	ATNR	2	0.38	0.54	0	0.61 (0.44, 0.74)	Fixed	0.71 (0.48, 0.95)	5.93	<0.01
		STNR	2	0.77	0.38	0	0.33 (0.11, 0.52)	Fixed	0.34 (0.11, 0.58)	2.85	<0.01
Primitive reflex test	INPP test	ATNR	1	Null	Null	Null	0.27 (0.00, 0.50)	Null	0.28 (0.00, 0.55)	1.96	0.05
	Schilder test		4	8.44	0.04	64.4	0.53 (0.41, 0.63)	Random	0.59 (0.44, 0.74)	7.53	<0.01
	INPP test	STNR	1	Null	Null	Null	0.38 (0.13, 0.59)	Null	0.40 (0.13, 0.68)	2.87	<0.01
	B-P test		3	2.04	0.36	2	0.40 (0.23, 0.55)	Fixed	0.42 (0.23, 0.61)	4.36	<0.01

INPP test, INPP Reflex Assessments Test; B-P test, Bender-Purdue Reflex Test.

Results from the four combined studies in the ATNR and anxiety group demonstrated a summary r value of 0.33 (*p* < 0.01), whereas those from the four combined studies in the STNR and anxiety group demonstrated a summary r value of 0.0007 (*p* < 0.01). Results revealed significant positive moderate correlation between ATNR and anxiety. However, no significant correlation between STNR and anxiety was found. The finding suggests that the higher the degree of ATNR non-integration, the more severe the anxiety symptoms.

Results from the five combined studies in the ATNR and impulsivity-hyperactivity group demonstrated a summary r value of 0.41 (*p* < 0.01), whereas those from the four combined studies in the STNR and impulsivity-hyperactivity group demonstrated a summary r value of 0.36 (*p* < 0.01). Results showed significant positive moderate correlation of ATNR and STNR with impulsivity-hyperactivity. The finding indicates that the higher the degree of ATNR and STNR non-integration, the more severe the impulsivity-hyperactivity symptoms.

Results from the five combined studies in the ATNR and learning problems group demonstrated a summary r value of 0.25 (*p* < 0.01), whereas those from the three combined studies in the STNR and learning problems demonstrated a summary r value of 0.43 (*p* < 0.01). Results revealed significant weak positive correlation between STNR and learning problems. This finding suggests that the higher the degree of ATNR and STNR non-integration, the more severe the learning problems.

Results from the four combined studies in the ATNR and perfectionism group demonstrated a summary r value of 0.39 (p < 0.01), whereas those from the three combined studies in the STNR and perfectionism group demonstrated a summary r value of 0.12 (*p* = 0.20). Results showed significant positive moderate correlation between ATNR and perfectionism. However, no significant correlation between STNR and perfectionism was found. The finding indicates that the higher the degree of ATNR non-integration, the more severe the perfectionism.

Results from the four combined studies in the ATNR and muscular tension group demonstrated a summary r value of 0.29 (*p* < 0.01), whereas those from the three combined studies in the STNR and muscular tension group demonstrated a summary r value of 0.15 (*p* = 0.28). Results revealed significant positive weak correlation between ATNR and muscular tension. However, no correlation between STNR and muscular tension was found. This finding indicates that the higher the degree of ATNR non-integration, the more severe the muscular tension.

Results from the three combined studies in the ATNR and psychosomatic difficulties group demonstrated a summary r value of 0.07 (*p* = 0.49), whereas those from the three combined studies in the STNR and psychosomatic difficulties group demonstrated a summary r value of 0.04 (*p* = 0.66). Results revealed no significant correlations between ATNR and STNR with psychosomatic difficulties.

Results from the three combined studies in the ATNR and antisocial behavior group demonstrated a summary r value of 0.23 (*p* = 0.01), whereas those from the three combined studies in the STNR and antisocial behavior group demonstrated a summary r value of 0.016 (*p* = 0.89). Results showed a significant positive weak correlation between ATNR and antisocial behavior, but no correlation between STNR and antisocial behavior was found. This finding suggests that the higher the degree of ATNR non-integration, the more severe the antisocial behavior.

#### 3.3.4. Subgroup analysis (sex)

According to sex, subgroup analysis results from the two combined studies in the ATNR and boys group demonstrated a summary r value of 0.23 (*p* = 0.03), whereas those from the two combined studies in the ATNR and girls group demonstrated a summary r value of 0.61 (*p* < 0.01). Results showed a significant positive weak correlation between ATNR and boys, whereas a significant positive strong correlation between ATNR and girls was found. The findings suggest that the higher the degree of ATNR non-integration, the higher the level of ADHD in girls than that in boys.

Results from the combined studies in the STNR and boys group demonstrated a summary *r* value of 0.44 (*p* < 0.01), whereas those from the combined studies in the STNR and girls group demonstrated a summary r value of 0.33 (*p* < 0.01). Results revealed significant positive moderate correlation between STNR with boys and girls. This finding indicates that the higher the degree of STNR non-integration, the higher the level of ADHD in boys than that in girls.

#### 3.3.5. Subgroup analysis (primitive reflex test)

According to the primitive reflex test, no subgroup analysis was conducted because of insufficient cases in the ATNR and Institute for Neuro-Physiological Psychology (INPP) Reflex Assessment Test group, and the summary r value was 0.27 (*p* = 0.05). Results from the four combined studies in the ATNR and Schilder test group demonstrated a summary r value of 0.53 (*p* < 0.01). Results showed a significant positive weak correlation between the ATNR and INPP Reflex Assessment Test group and a significant positive strong correlation between the ATNR and Schilder test group. The finding indicates that it is easier to measure the degree of non-integration of ATNR using the Schilder test compared with the INPP Reflex Assessments Test.

No subgroup analysis was conducted because of insufficient cases in the STNR and INPP Reflex Assessments Test group, and the summary r value was 0.38 (*p* = 0.05). Results from the four combined studies in the STNR and Bender–Purdue (B-P) Reflex Test group demonstrated a summary r value of 0.40 (*p* < 0.01). Results revealed significant positive moderate correlation of STNR with the INPP Reflex Assessments Test and B-*P* test groups.

Overall, findings from the sub-group analyzes show Conners-type scale, sex, and primitive reflex test could significantly moderate the relationship of ATNR and STNR primitive reflexes with ADHD.

**Figure 4:**
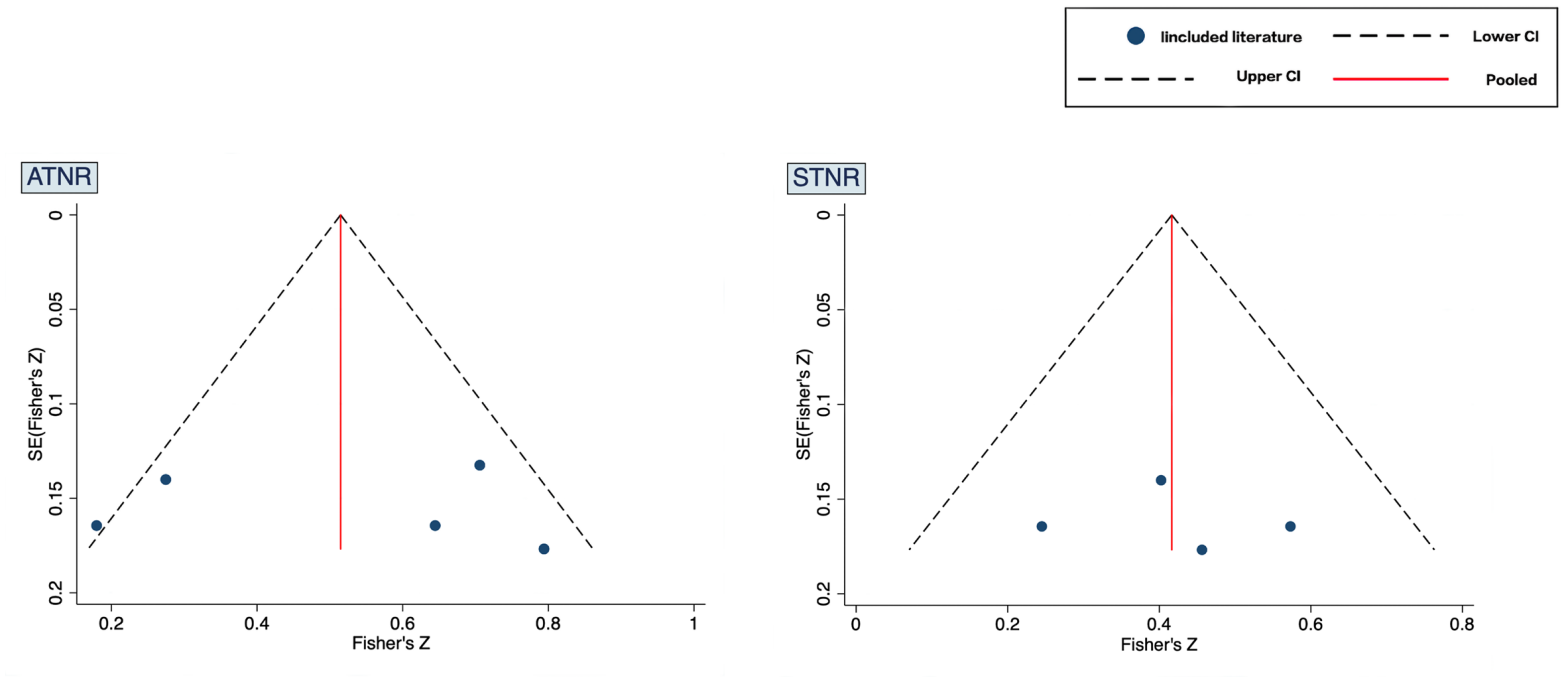
Funnel plot.

Publication bias was assessed using the Egger’s test (Figure 4). Results of studies on ADHD and ATNR demonstrated *t* = 0.12 and *p* = 0.91 (>0.05), whereas those of studies on ADHD and STNR demonstrated *t* = 0.19 and *p* = 0.87 (>0.05), both indicating no publication bias.

## 4. Discussion

We examined the correlation between primitive reflexes and ADHD. ATNR and STNR in the primitive reflexes demonstrated a significant positive and moderate correlation with ADHD; i.e., higher the degree of ATNR and STNR non-integration, more severe the ADHD symptoms in children.

The center of control of the primitive reflex originates from the brainstem (35). The primitive reflex of normally developed children gradually integrates into the limbic system of the brain (principally the hypothalamus, hippocampus, and amygdala), which is deeply connected with the cerebral cortex to jointly control the function of the brain. In contrast, if the primitive reflex is not integrated, the over-active brainstem still controls most of the movements and skills. This phenomenon inhibits the limbic system and cerebral cortex, which may lead to neurodevelopmental disorders (35–38). The limbic system controls emotion, memory, and learning. For example, the hypothalamus is central to maintaining a balance between emotional behavior, the amygdala participates in regulating emotional response, and the hippocampus is central to the regulation and control of emotional behavior, learning, and memory (38–42). Conversely, emotional behavior disorder causes persistent extraverted aggression, impulse, and hyperactivity in children, besides introverted fear, anxiety depression, and other behavior (43). In this study, ATNR was significantly correlated with the symptoms of conduct problems, anxiety, impulsivity-hyperactivity, and learning problems in children with ADHD. In contrast, STNR was not significantly correlated with anxiety, but was significantly correlated with the remaining problems. Thus, ATNR and STNR non-integration may lead to hyperactivity of the brainstem and inhibition of the limbic system, causing symptoms of conduct problems, anxiety, impulsivity-hyperactivity, and learning problems in children with ADHD.

According to MacLean’s trinity concept of brain and behavior (42) and Konicarova’s dissolution theory (44), the brain is divided into three levels (reptilian or primitive brain; limbic or mammalian brain; and neocortex, cerebrum, or human brain), each of which has different functions. The development of the central nervous system gradually develops from low-level discontinuous functions to high-level complete functions. Therefore, problems in the development of the lower level will block a higher stage of the nervous system. Further, functional disharmony or incompatibility among the three levels will lead to “functional dissociation,” which is related to the decline of cognitive and behavioral abilities in patients with ADHD. Moreover, the lack of neural connections at each level of the functional network under the trinity concept of brain and behavior will cause cognitive and behavioral problems. The reptilian or primitive brain causes hyperactivity, inattention, posture problems, uncoordinated movement, and inflexibility of fine movements; the limbic or mammalian brain can cause shyness, irritability, social boredom, and depression; and the neocortex, cerebrum, or human brain can cause dyslexia, writing disturbance, and logical reasoning problems. The reptilian or primitive brain is composed of the brainstem, pons, medulla oblongata, and cerebellum, and the control center of the primitive reflex originates from the brainstem (35). The failure of the primitive reflex to integrate within a reasonable time can be attributed to a lower-level developmental problem, which hinders the development of the advanced stage of the nervous system. Thus, a series of cognitive and behavioral problems exist. In this study, ATNR was significantly correlated with the symptoms of muscular tension, antisocial behavior, and perfectionism in children with ADHD, whereas STNR was not significantly correlated with antisocial behavior. Hence, we observed a significant positive correlation and causal relationship between primitive reflex and ADHD.

Women are more likely to have ADHD than are men; nonetheless, the severity of ADHD symptoms is higher in men than that in women (45–48). Therefore, we conducted a sex subgroup analysis of children with ADHD. Further, we performed a subgroup analysis of the primitive reflex measurement scale, which provided suggestions for the research design of the follow-up study. Despite a significant correlation between ATNR and ADHD in both boys and girls, we observed a strong correlation in girls and a weak correlation in boys. The correlation coefficients of STNR and ADHD were higher in boys than that in girls. From the perspective of neural mechanisms, sex differences in cerebellar resting activity may attribute to more severe motor impairment in boys with ADHD and poor performance in cognitive tasks in girls with ADHD. From the perspective of behavior and cognition, ATNR non-integration led to the impairment of eye-hand coordination skills and visual perception skills, which resulted in writing and reading difficulties in educational activities (7, 49). Further, STNR non-integration may lead to a decline in hand-eye coordination ability as well as poor posture and uncoordinated movement (7). Therefore, the correlation between ATNR non-integration and impaired visual cognitive ability is higher in girls with ADHD than that in boys. Conversely, the correlation between STNR non-integration and impaired motor ability is marginally higher in boys than that in girls. Regarding research design, the Schilder test results during ATNR measurements were higher than the INPP Reflex Assessments Test results, thereby necessitating attention toward the selection of scales in future research and the quality control of research.

### 4.1. Limitations

This study has some limitations. First, the reliability of the results may be affected by confounding factors, such as the limitations of included studies, inclusion criteria, and patient compliance. Second, a study of the relationship between primitive reflexes and ADHD is at the preliminary stage; therefore, this study cannot reveal the causal relationship between them. Future studies could further reveal the causal relationship through longitudinal research. Third, the included literature has limitations in discussing different types of primitive reflexes. In the future, researchers are required to discuss more about multiple types of primitive reflexes (4). Owing to the small number of documents included in this study, no further subgroup analysis of different subtypes and whether the participants were taking medications or not was conducted.

### 4.2. Conclusion

ATNR and STNR non-integration can lead to conduct problems, anxieties, imputivity-hyperactivity, and learning problems in children with ADHD; the primitive reflex and ADHD may have a causal relationship. The relationship between ATNR non-integration and impaired visual cognitive ability is higher in girls with ADHD than that in boys, whereas the relationship between STNR non-integration and impaired motor ability is marginally higher in boys with ADHD than that in girls. Regarding research design, we recommend the Schilder test to measure ATNR.

## Author contributions

MW contributed to the study design, data search, data analysis, and manuscript writing. JY participated in the study conceptualization and data search, collection, review, and analysis. AC and H-DK contributed to the data analysis and interpretation, provided a critical review of the manuscript, and participated in manuscript writing. All authors have read and approved the final version of the manuscript and agree with the order of presentation of the authors.

## Funding

This research was supported by the National Social Science Fund of China (21BTY096).

## Conflict of interest

The authors declare that the research was conducted in the absence of any commercial or financial relationships that could be construed as a potential conflict of interest.

## Publisher’s note

All claims expressed in this article are solely those of the authors and do not necessarily represent those of their affiliated organizations, or those of the publisher, the editors and the reviewers. Any product that may be evaluated in this article, or claim that may be made by its manufacturer, is not guaranteed or endorsed by the publisher.

## Footnotes

^1^ https://inplasy.com
